# Supplementary material for: Oxytocin functions as a spatiotemporal filter for excitatory synaptic inputs to VTA dopamine neurons
Source: eLife. 2018 Apr 20;7:e33892. doi: 10.7554/eLife.33892 (PMC5910020; doi:10.7554/eLife.33892)
Supplement: Transparent reporting form [file elife-33892-transrepform.docx]

***eLife’s* transparent reporting form**

We encourage authors to provide detailed information *within their submission* to facilitate the interpretation and replication of experiments. Authors can upload supporting documentation to indicate the use of appropriate reporting guidelines for health-related research (see [EQUATOR Network](http://www.equator-network.org/%20)), life science research (see the [BioSharing Information Resource](https://biosharing.org/)), or the [ARRIVE guidelines](http://www.plosbiology.org/article/info:doi/10.1371/journal.pbio.1000412) for reporting work involving animal research. Where applicable, authors should refer to any relevant reporting standards documents in this form.

If you have any questions, please consult our Journal Policies and/or contact us: [editorial@elifesciences.org](mailto:editorial@elifesciences.org).

**Sample-size estimation**

- You should state whether an appropriate sample size was computed when the study was being designed
- You should state the statistical method of sample size computation and any required assumptions
- If no explicit power analysis was used, you should describe how you decided what sample (replicate) size (number) to use

Please outline where this information can be found within the submission (e.g., sections or figure legends), or explain why this information doesn’t apply to your submission:

For all analyses in the manuscript, the number of neurons evaluated and the number of mice is reported, in the text and in the figure legends. On average, each electrophysiology comparison group included 9 neurons from 4.7 mice. Based on prior experience and power calculations, this is sufficient to detect differences below 20% with standard deviations typically observed in our data sets. For quantitative fluorescence *in situ* hybridization (FISH) assays, we used 1258 neurons from 3 mice. For combined retrograde labeling and FISH, 178-401 projection-specific neurons from 2-3 mice were evaluated, as reported in main text and legends.

**Replicates**

- You should report how often each experiment was performed
- You should include a definition of biological versus technical replication
- The data obtained should be provided and sufficient information should be provided to indicate the number of independent biological and/or technical replicates
- If you encountered any outliers, you should describe how these were handled
- Criteria for exclusion/inclusion of data should be clearly stated
- High-throughput sequence data should be uploaded before submission, with a private link for reviewers provided (these are available from both GEO and ArrayExpress)

Please outline where this information can be found within the submission (e.g., sections or figure legends), or explain why this information doesn’t apply to your submission:

The number of times each experiment was performed is clearly stated in the text. No outliers were excluded. Quality control for whole-cell voltage clamp recordings was pre-set at below ~20 MOhm series resistance and less than 10% change during the recording. Series resistance and input resistance were monitored using a 100 ms, 5 mV hyperpolarizing pulse at every sweep, and experiments were started after series resistance had stabilized

**Statistical reporting**

- Statistical analysis methods should be described and justified
- Raw data should be presented in figures whenever informative to do so (typically when N per group is less than 10)
- For each experiment, you should identify the statistical tests used, exact values of N, definitions of center, methods of multiple test correction, and dispersion and precision measures (e.g., mean, median, SD, SEM, confidence intervals; and, for the major substantive results, a measure of effect size (e.g., Pearson's r, Cohen's d)
- Report exact p-values wherever possible alongside the summary statistics and 95% confidence intervals. These should be reported for all key questions and not only when the p-value is less than 0.05.

Please outline where this information can be found within the submission (e.g., sections or figure legends), or explain why this information doesn’t apply to your submission:

Exact statistical methods used are stated for every comparison, with the p value reported as follows: the precise value is provided for all statistically non-significant comparisons, and the rest of p values are reported as *p<0.05, **p<0.01, ***p<0.001. All variance is presented as SEM. Paired tests were used when multiple conditions across the same neurons were analyzed, otherwise unpaired tests, as appropriate, were applied.

(For large datasets, or papers with a very large number of statistical tests, you may upload a single table file with tests, Ns, etc., with reference to sections in the manuscript.)

**Group allocation**

- Indicate how samples were allocated into experimental groups (in the case of clinical studies, please specify allocation to treatment method); if randomization was used, please also state if restricted randomization was applied
- Indicate if masking was used during group allocation, data collection and/or data analysis

Please outline where this information can be found within the submission (e.g., sections or figure legends), or explain why this information doesn’t apply to your submission:

Sex and age were balanced across groups. Beyond this, mice were randomly assigned to condition.

**Additional data files (“source data”)**

- We encourage you to upload relevant additional data files, such as numerical data that are represented as a graph in a figure, or as a summary table
- Where provided, these should be in the most useful format, and they can be uploaded as “Source data” files linked to a main figure or table
- Include model definition files including the full list of parameters used
- Include code used for data analysis (e.g., R, MatLab)
- Avoid stating that data files are “available upon request”

Please indicate the figures or tables for which source data files have been provided:

We are currently preparing readable, well-annotated matlab scripts for physiology and FISH analyses and will upload them to lab github account asap and link to this manuscript, if it is positively reviewed.
